# Silenced by design: epistemic injustice and older women’s experiences in total hip replacement

**DOI:** 10.1186/s12939-026-02796-0

**Published:** 2026-03-19

**Authors:** Lida Sarafraz

**Affiliations:** Medical Humanities Program, University of Texas at San Antonio, San Antonio, TX USA

## Abstract

Despite the increasing prevalence of total hip replacement (THR), older women’s post-operative experiences—particularly those related to pain, sexual wellbeing, and quality of life—remain insufficiently recognized within orthopaedic research and clinical practice. Drawing on bioethics and theories of epistemic injustice, this article offers a philosophical analysis of how gendered ageism shapes what counts as relevant knowledge in post-THR care. Building on Katrina Hutchison’s account of moral aggregation in medical device design, I argue that harms to older women arise not primarily through overt discrimination but through the cumulative effects of research design choices, outcome measures, and clinical norms that systematically marginalize experiential and age-specific concerns. To explain how these structural conditions translate into lived silencing within clinical encounters, I apply Kristie Dotson’s concept of testimonial smothering, showing how older women may reasonably withhold testimony about pain or sexual wellbeing in anticipation of misunderstanding, dismissal, or trivialization. Rather than presenting new empirical data, the article synthesizes existing research on orthopaedic outcomes, clinician–patient communication, and sexuality in later life to demonstrate why testimonial smothering in THR contexts is both plausible and ethically significant. Finally, drawing on José Medina’s notion of micro-resistance, the paper outlines modest, practice-level interventions that can mitigate epistemic exclusion within existing clinical and research environments. Attending to older women’s experiences in THR thus reveals broader structural challenges in how medical knowledge is produced, authorized, and acted upon in contexts of health equity.

## Introduction

Physical characteristics and clinical needs can vary significantly between men and women. However, these differences have often been overlooked in the design, testing, and evaluation of medical devices, with potentially harmful consequences for women patients. Analyses of orthopaedic implant design show that male bodies have frequently been treated as the default reference point, with limited attention to outcomes relevant to women’s wellbeing [1]. This pattern has contributed to persistent gaps in clinical evidence and interpretive resources for understanding women’s outcomes, shaping how risks, complications, and treatment success are identified in practice. Artificial hips used in total hip replacement (THR) provide a clear illustration of how gender-based exclusion from clinical research produces downstream effects. During THR (also known as total hip arthroplasty [THA]), damaged bone and cartilage are removed and replaced with prosthetic components (American Academy of Orthopaedic Surgeons [2]). Because these devices have historically been tested primarily on male bodies, women recipients are more likely to experience poorer outcomes, including chronic pain, reduced mobility, and complications affecting sexual function related to joint instability or misalignment [3].

After total hip replacement, sexual activity may be affected by pain, range-of-motion limitations, and concerns about joint stability or dislocation (e.g., [3]). However, sexual wellbeing is frequently treated as peripheral within orthopaedic care: it is commonly omitted from outcome measures and rarely discussed in pre- or post-operative counseling, particularly for older women patients [4, 5]. This omission is not merely a gap in guidance but reflects deeper assumptions about which concerns count as legitimate orthopaedic issues and whose embodied experiences are anticipated in clinical communication and device evaluation, illustrating how dominant biomedical framings systematically overlook key dimensions of patient experience. These risks are further compounded for older women—the primary population undergoing THR—whose anatomical, functional, and quality-of-life concerns are rarely foregrounded in device testing or outcome evaluation.

In this paper, I focus on sexual health communication after total hip replacement as a diagnostic case for examining how gendered ageism shapes orthopaedic knowledge practices more broadly—specifically, which outcomes are measured, which concerns are addressed in clinical settings, and whose experiences are treated as credible sources of insight. Methodologically, the article offers a philosophical analysis grounded in bioethics and the philosophy of medicine, informed by existing empirical research in orthopaedics, gerontology, and health communication. While the argument is theoretical, its aim is practical: to clarify how certain forms of harm arise in clinical and research contexts and to identify ethically meaningful points of intervention. In developing this analysis, I engage bioethics scholarship on gender bias in medical devices and clinical research, particularly accounts that frame these disparities as the result of cumulative or structural ethical failures. Bioethicists such as Katrina Hutchison argue that gender bias in medical device design and evaluation emerges through aggregative processes, in which individually minor decisions in research, regulation, and practice collectively produce systematic harm. Building on and critically extending this literature, I show how these ethical frameworks illuminate—but also leave underexplored—the age-based dimensions of exclusion that shape post-THR care.

To develop this argument, I draw on Kristie Dotson’s concept of *testimonial smothering* (2011) to explain how older women’s health concerns may be discouraged or silenced in post-THR contexts. I argue that gendered and age-based assumptions embedded in medical device design and healthcare interactions can make it difficult for older women to raise issues such as chronic pain or sexual wellbeing, even when these concerns significantly affect their quality of life. Importantly, this analysis does not claim to provide direct empirical proof that testimonial smothering occurs in every THR encounter. Rather, it shows how patterns documented elsewhere in medicine help explain why certain post-THR harms remain under-recognized and insufficiently addressed.

In the final section, I turn to possible responses. Drawing on José Medina’s concept of *micro-resistance*, I outline modest, practice-level interventions that can help counter these epistemic barriers. These proposals are offered not as comprehensive solutions but as illustrative strategies to improve clinical communication, recognize older women as credible knowers of their own bodies, and address persistent gaps in post-THR care.

Because the intended audience extends beyond philosophy to health equity scholars, clinicians, and policy-oriented readers, specialized philosophical terminology is used sparingly and explained where necessary. A brief terminology section follows to clarify key concepts before turning to their application in post-THR contexts.

### Scope and terminology

In this paper, I use the term *older women* to refer primarily to women aged 60 years and older, reflecting the age range in which total hip replacement is most commonly performed in the United States and other high-income settings. The analysis focuses in particular on women aged 65–85, while recognizing substantial heterogeneity within this group. Differences in health status, sexual activity, generational norms, and relationships to medical authority mean that the experiences of women in their early sixties may differ significantly from those of women in their eighties or nineties. Accordingly, this analysis does not presume uniform experiences among older women. Instead, it examines how structural patterns of gendered ageism shape the conditions under which sexual wellbeing becomes visible—or remains invisible—in post-THR care.

I also draw on the idea of *intersectionality* in a limited and practical sense. Rather than offering a theoretical account, I use the term to highlight how the intersections of age and gender interact to produce distinct forms of disadvantage. Older women are not affected by gender bias and age bias separately, but through their combination. This interaction helps explain why concerns that may be taken seriously for younger patients or for men—such as pain, mobility, or sexual wellbeing—can be overlooked or minimized for older women.

*Ggendered ageism* as an example of intersectionality, is the interaction of gender and age-based forms of marginalization that disproportionately disadvantage older women. In this “double jeopardy,” patriarchal norms intersect with a cultural preoccupation with youth, leading to a more rapid erosion of older women’s social credibility and moral standing than that of both younger women and older men ([6], p. 209).

Finally, the concept of *epistemic injustice* refers to harms that occur when individuals are wronged in their capacity as knowers—when their experiences, reports, or perspectives are given less credibility, taken less seriously, or excluded from decision-making. In healthcare settings, epistemic injustice can arise when patients’ accounts of their symptoms or priorities are discounted because of stereotypes about age, gender, or credibility. In this paper, epistemic injustice provides a framework for understanding how older women’s experiences—particularly those related to pain and sexual wellbeing after THR—can be systematically marginalized, even in the absence of overt discrimination or ill intent.

## Empirical context

### Gender, age, and post-THR outcomes

This section synthesizes existing research from orthopedic outcomes, clinical communication about sexuality in later life, and medical device regulation to provide the empirical background for the analysis that follows. Together, these literatures highlight recurring forms of gendered and ageist exclusion in post-THR care, motivating the subsequent philosophical analysis of epistemic injustice.

Biomechanical and motion-analysis studies show substantial sex differences in range of motion and joint stress during sexual activity. A detailed motion-capture study found that following total hip replacement, eleven of twelve sexual positions were considered safe for men, whereas only eight were safe for women [7]. Positions deemed safe for women were limited to those that avoided movements beyond the mechanical constraints of the artificial joint. Engaging in positions outside these limits can increase the risk of dislocation, edge loading, accelerated implant wear, and damage to the acetabular component. Related biomechanical analyses similarly indicate that certain sexual positions pose higher risks for some patients after THR, including women, depending on implant design and joint stability [3].

Moreover, a persistent lack of communication between orthopaedic surgeons or healthcare providers and women patients after total hip replacement—particularly regarding sexual activity—has been well documented. In a survey of members of the American Association of Hip and Knee Surgeons, 80% reported that they rarely or never discuss sexual activity with patients undergoing hip arthroplasty [8]. More recent prospective evidence confirms that sexual health remains largely absent from post-THR clinical discussions despite widespread patient interest. In a 2022 cohort study, 96% of patients reported that surgeons did not raise the issue of sexual activity, and more than three-quarters of sexually active patients indicated that they would have welcomed guidance [9]. This omission is reinforced by the fact that commonly used self-assessment questionnaires and traditional hip scoring systems do not include sexual activity as an outcome measure [10].

Studies focusing specifically on women highlight how this communication gap shapes patient behavior. In a study of 224 women who underwent total hip replacement, most reported preferring to receive information about post-operative sexual activity through the internet or written materials rather than directly from their surgeons, citing the perceived sensitivity of the topic ([11], p. 23–24). At the same time, many women expressed a strong desire to resume sexual activity after surgery and actively sought information about “safe” sexual positions [11]. Subsequent research suggests that this informational gap persists: a prospective multicenter study found that many patients reported unmet expectations regarding sexual activity after THR, reflecting limited counseling and guidance in clinical care [12]. As a result, patients may turn to informal or online sources, increasing the risk of exposure to misleading or poor-quality information about sexual activity following THR [13].

This dynamic is particularly evident in the context of total hip and knee replacement, procedures that are disproportionately performed on women in the United States, especially those aged 65–89, with a median patient age of approximately 67 years [14]. Evidence suggests that orthopaedic surgeons are generally reluctant to address sexual matters with patients after total hip replacement; when such conversations do occur, they are more often initiated with younger or married patients than with older women ([8], p. 238). This selective engagement reflects persistent cultural assumptions that older women are sexually inactive or uninterested in intimacy. Yet these assumptions are empirically unfounded.

Research on sexuality in later life shows that although women’s sexual practices may change with age—often due to physiological factors such as declining estrogen levels that can lead to vaginal dryness, atrophy, and discomfort during penetrative intercourse—sexual desire and intimacy remain important ([15], p. 249, [16]). Older women may engage less frequently in penetrative sex, but they continue to value and participate in a wide range of intimate practices, including affection, touch, and emotional closeness. For example, while only 13% of women aged 70 and older report regular sexual intercourse, 41% report frequent involvement in non-penetrative sexual activities such as hugging, kissing, caressing, and sexual touching (ibid. 247).

Importantly, lower rates of sexual activity among older women should not be attributed to diminished desire alone. Research shows that sexual engagement in later life is often shaped by structural and relational factors such as widowhood, partner illness, caregiving responsibilities, or limited opportunities for intimate connection [17]. Changes in social context—such as forming new partnerships or entering supportive living environments—can be associated with renewed sexual and intimate engagement [18]. These findings undermine assumptions that sexual wellbeing is irrelevant to older women’s lives and help explain why its routine exclusion from orthopaedic care reflects gendered ageism rather than patient disinterest.

Orthopaedic research has long documented significant sex differences in clinical outcomes [19]. For example, hormonal changes during menstrual cycles and pregnancy can affect knee joint laxity in women [20]. As Templeton [21] observes, orthopaedic studies have often conflated sex and gender or treated them as secondary factors rather than as concepts that shape research questions, study design, or outcome selection. Even when women are included in study populations, sex-based inclusion does not reliably translate into sex- or gender-sensitive analysis; research frequently relies on standardized models and outcome measures derived from male bodies and male-typical experiences (Merone et al., 2022). Treating sex and gender as background variables matters because it shapes what researchers attend to and what clinicians come to see as clinically relevant. When these factors are marginalized, aspects of patient experience such as sexual wellbeing, chronic pain, and age-related functional needs are more likely to be overlooked in device design, outcome measurement, and clinical care.

To sum up, this literature shows that post-THR care is shaped by overlapping forms of gender- and age-based exclusion across clinical communication, research design, outcome measurement, and regulatory evaluation. Orthopaedic outcomes research documents sex- and age-specific risks that prevailing care models poorly capture, while studies of patient–provider communication reveal persistent gaps in addressing sexual wellbeing for older women despite clear relevance to quality of life. At the same time, research and regulatory practices continue to marginalize older women through age-restricted trials, sex-insensitive analyses, and evaluative frameworks that privilege technical performance over lived effectiveness. These patterns create the empirical conditions under which older women’s post-THR experiences become epistemically peripheral, motivating the philosophical analysis that follows.

### Structural features of orthopaedic research and knowledge production

Whereas the previous section documented sex- and age-based disparities in post-THR outcomes and communication, this section explains why such gaps persist across orthopaedic research and device development. The central claim is not that orthopaedic clinicians or researchers are indifferent to patients’ experiences, but that the organization of orthopaedic research systematically deprioritizes experiential, gendered, and age-specific forms of knowledge. As a result, certain patient concerns—particularly those related to sexual wellbeing and quality of life—remain marginal within dominant research and clinical frameworks.

Age bias further compounds these limitations. Early evidence showed that older adults were routinely and unjustifiably excluded from clinical research, often through arbitrary upper age limits that ethics review committees failed to challenge [22]. More recent analyses demonstrate that this pattern not only persists but is structurally embedded in contemporary research practices. Reviewing interventional trials registered on ClinicalTrials.gov between 2010 and 2021, Nguyen, Mika, and Ninh [23] show that upper age caps remain widespread across major disease areas, including cardiovascular disease, and that the implementation of the NIH Inclusion Across the Lifespan Policy has not produced a meaningful reduction in age-based exclusions [24].

Complementing this trial-level evidence, a systematic analysis of FDA initial approval documents reveals that older adults remain underrepresented even at the point of regulatory authorization. Although pharmacokinetic data specific to older populations have become more common over time, safety and efficacy data for elderly patients remain absent in the majority of approval documents, with no significant improvement across decades [25]. Together, these findings indicate that age-based exclusion is not merely a legacy problem but an ongoing epistemic failure that limits the external validity of clinical evidence for conditions disproportionately affecting older adults—and, by extension, shapes the knowledge base informing post-operative and post-device care.

Although age-related physical and hormonal differences are well documented in orthopedic research, they are rarely incorporated meaningfully into study design or analysis. As Beck and colleagues observe, orthopedic studies typically aggregate wide age ranges into single cohorts, reporting average ages with standard deviations rather than analyzing age-specific outcomes. Patient samples often span from adolescence to late adulthood, yet statistical analyses seldom disaggregate outcomes for older patients, particularly those over 55, due to concerns about sample size and statistical power ([26]; e480).

Recent systematic reviews of orthopaedic and geriatric orthopaedic research show that although disparities research has increased sharply since 2019, it remains overwhelmingly retrospective, focused on surgical or utilization outcomes, and largely inattentive to patient-centered experiences such as sexuality, quality of life, disability, and social meaning [27, 28]. Within this literature, arthroplasty—particularly total hip replacement—emerges as a salient site for examining how clinical outcomes, patient communication, and device design intersect, making it a revealing case for analyzing the epistemic marginalization of older women’s experiences.

Broader regulatory and research priorities within the medical device landscape reinforce these assumptions. Scholars of health research governance argue that medical device evaluation has historically emphasized *performance*—how well a device functions according to technical specifications—over *effectiveness*, understood as therapeutic benefit in real-world clinical contexts, including patient experience [29]. Although this analysis does not provide THR-specific outcome data, it highlights how regulatory frameworks shape what kinds of evidence are generated, valued, and translated into care, forming a crucial backdrop for post-THR knowledge production.

These regulatory priorities intersect with longstanding patterns in biomedical research design. Studies of pain research and device development suggest that male bodies have often been treated as the default reference point, embedding gendered assumptions about bodily norms and pain perception into research protocols. Such assumptions have been linked to delayed recognition of device-related harms and persistent gaps in sex-specific evidence [1]. Within orthopaedics, this contributes to limited attention to outcomes that disproportionately affect women, including chronic pain trajectories, recovery experiences, and sexual wellbeing.

Structural features of the orthopaedic workforce further constrain knowledge production. Heavy clinical workloads, procedural incentives, and limited institutional support for research characterize orthopaedic surgery. As a result, only a small proportion of surgeons engage in sustained academic research [30]. Even among physician–scientists, orthopaedics remains an outlier: national data show that MD–PhDs are far less likely to train in orthopaedic surgery and, when they do, are less likely to hold full-time academic appointments or devote substantial time to research [31].

Gender disparities compound these constraints. Women constitute only a small fraction of arthroplasty surgeons, resulting in limited gender diversity within both clinical practice and academic leadership. This underrepresentation matters not merely as a demographic imbalance, but also because it shapes which research questions are perceived as important and which patient experiences are considered salient [32]. Empirical studies indicate that women surgeons face structural barriers to research participation, including exclusionary professional cultures and work–life pressures specific to surgical specialties [33]. Within an already procedurally oriented field, these dynamics further narrow attention to issues that disproportionately affect women patients.

Funding structures reinforce these patterns. Orthopaedic research—particularly patient-centered and experiential research—has long been underfunded by public agencies. Between 1985 and 2021, fewer than one in twenty academic orthopaedic surgeons received NIH funding [34, 35]. Arthroplasty research remains especially underfunded despite its clinical prevalence, and women are dramatically underrepresented among principal investigators of hip and knee arthroplasty trials [36]. In this context, industry funding plays a dominant role in shaping research agendas, often privileging device optimization and regulatory approval over long-term, qualitative, or gender- and age-sensitive outcomes.

These dynamics help explain why biomedical engineers and device manufacturers frequently lead orthopaedic innovation, while surgeons are positioned primarily as implementers rather than co-producers of knowledge. Intellectual property regimes, regulatory constraints, and institutional incentives further limit sustained collaboration among patients, clinicians, and researchers. As Hutchison argues, these physical and epistemic barriers systematically distance patients—especially women—from the sites of device knowledge production, with high epistemic costs [1].

These institutional features help explain why sex- and age-based disparities in post-THR knowledge persist across research, device design, and clinical pathways. Constraints on time, funding, workforce composition, and evaluative standards narrow the questions asked and the outcomes treated as clinically meaningful. Within this structure, experiential dimensions of care—such as sexual wellbeing, chronic pain, and quality of life—are especially likely to be sidelined. The next section turns to the ethical frameworks needed to conceptualize these structural patterns as forms of epistemic injustice rather than isolated failures of communication or intent.

## The limitations of previous ethical approaches

Bioethics scholars have examined disparities in medical device design from several ethical perspectives. Katrina Hutchison [1] frames these disparities as a *moral aggregation problem*, arguing that small, seemingly harmless decisions in device development and evaluation can accumulate into significant harm ([1], p. 577). By contrast, Liao and Carbonell [37] offer a more explicitly structural account, arguing that biased medical devices function as tools of oppression that reinforce existing social inequalities affecting women and other marginalized groups. While both approaches offer important insights into how bias becomes embedded in medical technologies, I argue that they remain limited in their ability to capture the epistemic and age-related dimensions of harm experienced by older women.

Hutchison’s analysis draws on the concept of aggregative harm, originally articulated by Elizabeth Kahn [38]. Kahn describes aggregative harm as arising from the cumulative effects of many individual actions, none of which is necessarily harmful on its own. Her well-known example is air pollution: a single person driving a car does not cause noticeable harm, but the combined effects of many drivers produce serious environmental damage. This framework is particularly useful for cases in which harm becomes visible only at the population level, rather than through isolated acts of wrongdoing. Applying this model to medical devices, Hutchison argues that practices such as recruiting predominantly male participants or failing to analyze sex-specific outcomes may appear benign in isolation but can generate substantial harm once they reach a tipping point ([1], p. 577).

In the context of medical device design, such harm is often detectable only through large-scale patterns, prompting attention to systemic bias rather than individual fault. Hutchison emphasizes that gender bias emerges across multiple levels of practice, including research design, engineering standards, and regulatory oversight. For example, biomedical engineers rarely interact directly with patients, which can result in design norms that implicitly treat male bodies as standard and female bodies as deviations ([1], p. 580). On this account, bias is not reducible to prejudice or negligence but reflects layered institutional processes.

For this reason, Hutchison argues that top-down regulatory efforts—such as FDA policies mandating greater inclusivity—are necessary but insufficient. She proposes a multilevel response that combines regulatory reform with bottom-up strategies, including education about implicit bias among clinicians, engineers, and researchers. Addressing gender disparities, she argues, requires coordinated efforts across individual and institutional domains, since no single actor can be held solely responsible for cumulative harm ([1], p. 586).

Building on this framework, Liao and Carbonell (2023) advance what they call an *aggregated solutions approach*. Whereas Hutchison emphasizes how dispersed actions generate harm, Liao and Carbonell focus on the resilience of oppressive systems themselves. They argue that because oppression is structurally entrenched, it can only be dismantled through multiple, overlapping interventions rather than isolated reforms. Their proposal calls for coordinated “redundant anti-oppressive interventions” to identify and remove biased medical tools from the systems that sustain them (Liao and Carbonell 2023, 19–20). Although they acknowledge the difficulty of coordination across institutions, they argue that layered interventions are necessary precisely because oppression adapts and persists over time.

Despite their differences, both frameworks share an important assumption: that individual biased actions—such as designing a study around male participants—are ethically limited in their impact when considered in isolation. While Hutchison treats such actions as separately harmless until they aggregate into systemic harm, Sarafraz [39] argues that even a single male-biased study can be epistemically and clinically consequential. Once published, such studies are often generalized, cited, and built upon, shaping future research agendas, clinical assumptions, and patient care in ways that disadvantage women.

A clear illustration of this dynamic is the widely circulated motion-capture study discussed in Section “Empirical context”, which examined the safety of twelve sexual positions following total hip replacement. By the end of 2025, visualizations of this study had been viewed more than 19 million times on YouTube and cited across both clinical and academic contexts (Artanim Foundation [40]). Its influence extends beyond clinical guidance to ethical and philosophical discussions; Hutchison herself cites the study as empirical evidence supporting claims about the risks of sexual activity for women after THR.

Yet the study relies on young, heterosexual couples aged 26–31 as biomechanical models, thereby establishing a normative standard that does not reflect the bodies, practices, or priorities of older patients ([7], p. 640). In addition, the study defines sexuality exclusively in terms of penetrative intercourse, despite substantial evidence that many older women prioritize other forms of intimacy. This case illustrates how a single, highly visible study can shape clinical expectations and ethical discourse while systematically excluding the lived experiences of those most affected. When such research is repeatedly cited and treated as authoritative, its limitations become epistemically consequential rather than merely methodological.

Although frameworks of moral aggregation and coordinated institutional reform help explain how bias becomes embedded in medical device design, both remain incomplete insofar as they give insufficient attention to age as a distinct source of disadvantage. By focusing primarily on gender while leaving age implicit, these accounts risk promoting solutions that disproportionately benefit younger women while overlooking the needs of older women. Yet older women differ from younger patients in anatomy, physiology, pain trajectories, sexual wellbeing, and communication needs. Ignoring age alongside gender, therefore, limits both the explanatory reach and the corrective potential of these ethical approaches.

Moreover, because these frameworks prioritize population-level patterns and collective responsibility, they risk obscuring how harm is experienced at the individual patient level—particularly among those whose lives do not align with dominant clinical narratives. Addressing injustice in this context requires attention not only to abstract structures but also to patients’ accounts of pain, recovery, and everyday functioning.

These limitations are reinforced by older women’s marginal position within medical research hierarchies. When study designs fail to anticipate age-specific bodies or practices, older women’s experiences are less likely to be captured or interpreted as clinically meaningful. Silence or limited evidence may then be misread as confirmation of preexisting assumptions about older women’s lives rather than as a product of exclusion.

## Testimonial smothering as a theoretical tool

Framing these patterns as forms of epistemic injustice helps clarify whose knowledge is recognized in healthcare and whose experiences shape clinical understanding. As Miranda Fricker defines it, epistemic injustice occurs when someone is wronged specifically in their capacity as a knower ([41], p. 20). Philosophers of medicine have shown that such injustices are not incidental but structurally embedded in contemporary healthcare systems. Carel and Kidd [42, 43], for example, argue that medical institutions are organized around epistemic asymmetries that privilege professional expertise while systematically marginalizing patient experience, particularly in contexts of chronic illness. These asymmetries limit patients’ epistemic agency by shaping what counts as relevant information, discouraging the articulation of lived experience, and prioritizing standardized, quantitative indicators over qualitative accounts of illness. Crucially, these patterns are sustained not simply by individual clinician bias, but by institutional norms, time pressures, and evidentiary standards that structure everyday clinical practice [43].

While Carel and Kidd illuminate the structural dimensions of epistemic injustice in healthcare, Kristie Dotson’s concept of testimonial smothering offers a more fine-grained account of how such harms are enacted and anticipated within individual interactions. Dotson introduces testimonial smothering to describe situations in which speakers truncate or withhold their testimony because they reasonably anticipate that their audience lacks the willingness or capacity to understand them. She defines testimonial smothering as “the truncating of one’s own testimony in order to ensure that the testimony contains only content for which one’s audience demonstrates testimonial competence” ([44], p. 244). In practical terms, this form of self-silencing occurs when individuals adjust what they say—or refrain from speaking altogether—because they expect misunderstanding, dismissal, or discomfort from those listening.

In healthcare settings, testimonial smothering does not require overt hostility or explicit exclusion. It can arise in routine clinical encounters when patients come to perceive certain concerns as inappropriate, irrelevant, or unlikely to be taken seriously. In post–total hip replacement care, older women are particularly vulnerable to this form of epistemic injustice when their experiences—especially those related to pain, recovery, or sexual wellbeing—are anticipated as marginal or outside the scope of legitimate orthopaedic concern. Rather than treating these patterns as isolated failures of communication or gaps in evidence, the following section examines how structural features of post-THR care shape the epistemic conditions under which testimony is voiced, constrained, or silenced, with particular consequences for older women.

Dotson outlines three conditions that foster testimonial smothering:

1. The content of the testimony is risky or "unsafe";
2. The audience displays testimonial incompetence regarding the content; and
3. This incompetence stems from pernicious ignorance.

Dotson defines unsafe testimony as statements that “run the risk of leading to the formation of false beliefs that can cause social, political, and/or material harm” ([44], p. 244). One of her central examples involves a person of color who withholds information about her community’s experiences of racism during an interaction with an interlocutor who responds with sarcasm or a dismissive tone. In this context, the speaker judges that continuing the conversation would be unproductive, given that the audience does not appear willing or able to understand her testimony.

In such microaggressive encounters, persisting in the conversation may be epistemically unsafe, insofar as it risks reinforcing distorted or dismissive interpretations of lived experience. Dotson argues that these reactions signal the audience’s testimonial incompetence regarding race-related testimony, grounded in a lack of racial sensitivity rather than in any failure on the part of the speaker.

The second condition of testimonial smothering, testimonial incompetence, refers to situations in which the audience lacks either the capacity or the willingness to adequately understand the speaker’s testimony ([44], p. 246). Engaging such an audience—particularly on topics like structural racism—often exposes the speaker to repeated invalidation and increased emotional labor. In these circumstances, continuing the exchange is unlikely to be productive. As a result, the speaker may choose to withhold or limit their testimony, engaging in self-silencing as a protective response rather than as a sign of epistemic failure.

Finally, pernicious ignorance, the third condition for testimonial smothering, refers to a form of ignorance that is especially harmful and persistent because social power relations sustain it ([44], p. 250). Dotson uses the term situated ignorance to describe how individuals’ dominant social or epistemic positions can generate this kind of ignorance. In such cases, an audience member may not intentionally dismiss or misunderstand a speaker’s testimony; rather, their social location and lived experience can limit what they are able—or willing—to recognize as meaningful knowledge ([44], p. 248).

Situated ignorance is pernicious because of the cumulative harms it imposes on speakers. These harms include the emotional labor involved in navigating invalidating exchanges, the psychological work required to make sense of being misunderstood or dismissed, and concerns about stigma, marginalization, or material consequences tied to how one’s testimony may be received. Faced with these risks, speakers may reasonably judge that speaking is unsafe or futile. For Dotson, the convergence of unsafe testimony, testimonial incompetence, and pernicious ignorance is often sufficient to prompt self-silencing and the truncation of testimony ([44], p. 249).

We can now illustrate testimonial smothering in the context of older women following total hip replacement. Testimonial smothering arises when older women anticipate that sharing certain experiences—such as ongoing sexual activity, sexual concerns, or persistent post-surgical pain—will be misunderstood, minimized, or dismissed by their clinicians. In orthopaedic care, this risk is shaped in part by a pronounced social and experiential gap between patients and providers. With only about 2% of arthroplasty surgeons in the United States being women, most older women patients receive care from male surgeons whose clinical training and lived experience may differ substantially from their own ([33], p. 527). Anticipating limited understanding or receptivity, older women may reasonably choose to withhold aspects of their experience, thereby reinforcing silence around their specific needs.

For older women post-THR, discussions of sexual activity are especially likely to be perceived as risky. Persistent cultural stereotypes portray older women as asexual or uninterested in intimacy, and these assumptions can subtly shape clinical encounters. When patients sense that such beliefs are operative—whether explicitly or implicitly—they may fear judgment, awkwardness, or dismissal if they raise sexual concerns. In these circumstances, self-censorship becomes a protective strategy, consistent with Dotson’s account of testimonial smothering, even when the information withheld is clinically relevant to recovery and quality of life.

Importantly, the relevance of gender in these encounters should not be understood as a simple matter of gender concordance between patient and clinician. Rather, empirical research suggests that older patients’ willingness to disclose sexual concerns depends largely on whether they anticipate that their testimony will be taken seriously within the clinical encounter. Studies show that physicians rarely initiate conversations about sexuality with older adults and often treat sexuality in later life as peripheral to medical care. In this context, patients learn—implicitly or explicitly—that such concerns are unlikely to be welcomed or addressed, leading many to refrain from raising them themselves [45].

Evidence from a nationally representative U.S. sample similarly shows that while many adults aged 65–80 consider sex important to their quality of life, fewer than one in five had discussed sexual health with a healthcare provider in the previous two years. When such conversations did occur, they were most often initiated by patients rather than clinicians [46]. Notably, older women were significantly less likely than older men to report these discussions, despite expressing interest in sex and sexual satisfaction. This pattern suggests that older women are not only less likely to be invited into conversations about sexual health but may also anticipate dismissal or discomfort, reinforcing patterns of self-censorship within clinical encounters.

In this context, the male-dominated nature of orthopedic surgery may function as one structural cue—among others—shaping older women’s expectations about whether their testimony will be taken seriously. Age differences, generational norms surrounding sexuality, and the authority of surgical expertise can further intensify anticipations of misunderstanding, trivialization, or dismissal. Silence around sexual wellbeing after THR is therefore better understood as a response to anticipated testimonial incompetence than as a lack of willingness to speak.

Importantly, the relevance of gender in these encounters should not be understood as a simple matter of gender concordance between patient and clinician. Empirical research suggests instead that older patients’ willingness to disclose sexual concerns depends on whether they anticipate their testimony will be treated as legitimate and intelligible within the clinical encounter. Studies of physician–patient communication show that clinicians rarely initiate conversations about sexuality with older adults and often regard sexuality in later life as peripheral to medical care (Levkovich et al. 2021). Clinicians without training in human sexuality are particularly unlikely to invite such discussions, reinforcing patterns of silence that reflect reasonable expectations of dismissal rather than failures of patient engagement.

Taken together, these dynamics suggest that older women’s silence about sexual wellbeing after total hip replacement is not best understood as individual reticence or lack of interest. Rather, it reflects a structurally situated response to anticipated testimonial incompetence within clinical encounters shaped by gendered and ageist assumptions. In this context, withholding testimony can be a rational and protective strategy rather than a failure of patient engagement.

Framing this pattern as a case of testimonial smothering—a form of epistemic injustice in which speakers truncate their own testimony in response to unsafe conditions of uptake—helps clarify how empirical disparities in research design, clinical communication, and professional culture translate into lived silencing. Importantly, this analysis highlights a limitation of existing ethical approaches to biased medical devices. While frameworks focused on moral aggregation or anti-oppressive interventions capture important structural features, they largely treat gender as a single axis of concern and risk overlooking how age compounds epistemic vulnerability.

This article does not claim to offer direct empirical evidence that testimonial smothering occurs in every post-THR clinical encounter involving older women. Rather, drawing on established patterns of gendered and age-based disparities in orthopaedic research and care, well-documented communication gaps around sexuality in later life, and persistent patient-reported harms that remain insufficiently addressed, it advances testimonial smothering as a plausible and under-recognized mechanism shaping older women’s post-THR experiences. Even in the absence of condition-specific confirmation, the ethical costs of ignoring this risk—particularly for a population subject to layered marginalization—justify closer attention in both research and clinical practice.

## Practical applications and solutions

Philosopher Medina [47] introduces the concept of *micro-resistance* to describe everyday practices that respond to epistemic harms such as testimonial smothering and microaggressions. This framework is especially useful for thinking about how the silencing of older women in post–total hip replacement (THR) contexts might be challenged, even in the absence of immediate structural or regulatory reform. Rather than focusing exclusively on large-scale institutional change, Medina directs attention to small, situated actions that can interrupt patterns of epistemic exclusion as they occur.

Micro-aggressions call for micro-resistance, for micro-practices of resistance in which the insidious aggressive moves with disabling effects are neutralized, halted, or at least weakened. Micropractices of resistance are heavily situated and contextualized moves that often require a lot of inventiveness and cunning as well as other epistemic virtues such as courage.[…]micro-activities of resistance taken collectively can help to undermine, weaken, and ultimately destroy such culture and to create one of support and mutual protection instead, even if no single act of micro-resistance will achieve that by itself.[…] acts of micro-resistance do not need to be issued necessarily by the person suffering the epistemic violence of the micro-aggression, but it can be produced effectively (sometimes even more effectively) by others involved in the interaction even though they were not targeted, and in some cases even by bystanders and eavesdroppers.[…] Micro-practices of resistance can also work preventively, for example by proactively offering gestures of validation to members of a group that has been stigmatized or is likely to encounter micro-invalidations in a particular context ([47], pp. 258-9).

This account highlights how epistemic injustice can be addressed through ordinary professional practices rather than only through policy change or formal regulation. Small interventions—such as explicitly inviting discussion of stigmatized topics, responding seriously to patient concerns that are often minimized, or countering ageist assumptions in clinical training—can reduce the conditions that enable testimonial smothering. Because these practices can be undertaken by clinicians and institutional actors who are not themselves the targets of silencing, micro-resistance provides a practical and ethically tractable framework for improving epistemic inclusion within existing care structures.

If testimonial smothering plausibly operates in post-THR contexts, then such responses are ethically warranted. Several examples of micro-resistance are particularly relevant to orthopaedic care.

First, media representation can function as a form of micro-resistance by amplifying older women’s voices and normalizing later-life sexuality. Popular and digital media—such as television series, podcasts, social media campaigns, and graphic medicine—can challenge ageist assumptions about intimacy after surgery and provide alternative narratives that affirm older women’s experiences as ordinary and legitimate.

Second, traditional educational resources can lower the threshold for conversations that patients may otherwise self-censor. Clear, accessible materials—such as booklets designed to facilitate discussion among healthcare providers, patients, and partners—can serve as a shared reference point for raising sensitive topics, particularly for patients who do not rely on digital technologies ([48], p. 193).

Third, medical training and language development are crucial. The absence of shared clinical language for later-life sexuality—especially forms of intimacy that do not center on penetrative intercourse—contributes to its exclusion from clinical encounters. When older women’s sexual practices do not align with youth-centered or heteronormative models, both patients and clinicians may lack the conceptual resources needed to name and discuss relevant concerns. Developing more inclusive descriptive frameworks, alongside targeted training in medical education and continuing professional development, could help legitimize these topics within research and care. Bioethicists are well positioned to contribute to such efforts by facilitating reflection on age- and gender-based epistemic bias and supporting curriculum development.

While the structural constraints discussed earlier in the paper underscore the importance of top-down remedies—such as funding reform, regulatory change, and institutional incentives—they do not preclude more localized forms of resistance. Drawing on Medina’s account of micro-resistance, it is possible to identify modest but meaningful practices through which entrenched epistemic patterns might be challenged from within orthopaedic research, training, and clinical environments. These include advocating for patient representation in device development, promoting research agendas attentive to age- and gender-specific outcomes, and questioning the male-dominated norms that shape orthopaedic education and professional culture.

Taken together, these interventions illustrate how micro-resistance can operate alongside broader institutional reform to disrupt epistemic patterns that marginalize older women in post-THR care. Although limited in scope, such practices matter precisely because they intervene at the sites where testimonial smothering occurs: everyday clinical interactions, educational settings, and knowledge-making practices. Attending to these sites clarifies how epistemic injustice in orthopaedic medicine can be challenged incrementally, without presuming immediate structural transformation, while still keeping the need for such transformation firmly in view.

## Data Availability

No datasets were generated or analysed during the current study.
